# “*What are you doing here*?”: Narratives of border crossings among diverse Afghans going to the UK at different times

**DOI:** 10.3389/fsoc.2023.1087030

**Published:** 2023-02-01

**Authors:** María López, Louise Ryan

**Affiliations:** Global Diversities and Inequalities Research Centre, London Metropolitan University, London, United Kingdom

**Keywords:** migration journey, narratives of migration, hostile environment, border crossing, Afghans in the UK

## Abstract

Through the “hostile environment” migration policy, the UK government has expressed its commitment to do whatever possible to deter and expel unwanted migrants. Faced with the loss of power in the context of globalization, the Conservative administration, elected in 2010, presented itself as a guarantor of citizens' security. The political discourse of “taking back control” of the nation's borders has resulted in increasingly restrictive immigration and asylum policies. In this paper, we present narratives of Afghans who arrived in the UK at different times and through different routes. As well as those evacuated from Kabul airport in 2021, we also interviewed participants who traveled *via* insecure routes over land and sea often taking months, or even years, and involving expensive people smugglers. While the evacuation from Kabul was a very public and highly reported event, often with celebratory tones in the international media as Western governments sought to “rescue” Afghan allies, those Afghans who travel to the UK *via* illegal routes are often stigmatized; demonized in press and political discourses. Building on the emerging body of literature that uses “journey as a narrative device” and drawing upon our novel dataset, we analyze how diverse migrants tell their stories and present agency, within contexts of extreme hazards, to achieve their imagined future. Moreover, applying a spatio-temporal lens we advance understanding of the intersection of place and time in how Afghans traveling to the UK, including recent evacuees, are framed differently with resultant consequences for how border crossings are negotiated and narrated. In so doing, we complicate simplistic categories of deserving vs. undeserving, genuine vs. fraudulent, evacuees vs. irregularised migrants.

## Introduction

The announcement by US President Joe Biden, in April 2021, of withdrawing US troops from Afghanistan triggered a race against time for Western governments to leave ahead of 31st August. The Taliban's rapid advance throughout the country and their national power takeover on 15th August turned Kabul Hamid Karzai International Airport into the epicenter of a precarious international operation that aimed to evacuate those who worked with the government, their families and other vulnerable people on humanitarian flights (UNOCHA, 2022).

Thousands of civilians packed into the area around the airport, waiting for days in appalling conditions, enduring high temperatures without water or food (Latifi, 2021). During these critical days, the Taliban fired shots into the air and tried to disperse people with batons, killing at least 20 people (Sabbagh et al., 2021). A bomb attack by Islamic State took place at the airport on 26th August, killing more than 72 civilians and 13 US personnel (Sullivan, 2021). Desperation and panic ensued, with apparent fatalities after people were seen clinging to moving US aircraft (Harding and Doherty, 2021). On the 31st August, the Taliban celebrations of the departure of the Western governments with fireworks contrasted with the desolation of the hundreds of civilians still at the airport who had not managed to leave the country (Burns and Baldora, 2021).

The British Army evacuated around 15,000 Afghans. In the UK, the government received these evacuees as part of Operation Warm Welcome, which aimed to save the lives of those who worked with and for the British forces in Afghanistan. By ensuring their safe departure, the government intended to fulfill a moral debt to them and “ensure Afghans arriving in the UK receive the vital support they need to rebuild their lives, find work, pursue education and integrate into their local communities” (Gov.UK, 2021).

The welcoming discourse toward Afghan evacuees contrasted with parallel discourses about Afghans arriving on English coasts *via* irregular routes, e.g. in small boats or the backs of lorries. In 2021, more than 25,700 people crossed the English Channel in small boats—more than triple the 2020 total. Afghans constituted 24% (1,094) of the 4,540 people who arrived on English shores in small boats between January and March 2022, followed by Iranians (16%; 722) and Iraqis (15%; 681) (ITV, 2022). In British policy and media discourses these arrivals are presented on the one hand as a threat to the UK's security and welfare systems and, on the other hand, as victims of human traffickers who charge exorbitant prices and use intimidating tactics.

As noted by Schapendonk et al. (2021), the division of people into particular migrant categories is a “normative artefact” created by migration policies. The fact of crossing borders in different ways, regular vs. irregular, is used as a rationale for different treatment upon arrival in the UK. Hence, through different policies toward Afghans who arrive *via* different routes, the British government has created an artificial distinction between those who are fleeing the same conflict at the same time. Immigration policies that seek to differentiate between different types of migrants arriving *via* different routes “perpetuate and reinforce a simplistic dichotomy” between the “genuine” and the “fraudulent” and between the deserving and the undeserving in accessing the welfare system (Karyotis et al., 2021, p. 483). Indeed, in the UK since the early 2000s, successive governments have “separated asylum seekers and refugees, with the former considered unwanted and treated with suspicion and the latter reluctantly accepted” (Karyotis et al., 484). In this regard, El-Enany (2020) talks about “irregularised” (rather than “irregular”) migrants to emphasize the government's efforts to silence and marginalize those arriving *via* irregular routes, which is reminiscent of past colonial times (El-Enany, 2020).

In this article, we engage with recent theories around “migrant journey” (Gough and Gough, 2019; Amrith, 2021; Crawley and Jones, 2021; Schapendonk et al., 2021). Using journey narratives as an interpretative device (Mason, 2004; Kaytaz, 2016), we apply a spatio-temporal lens (Erel and Ryan, 2019) to understand how migrants' journey narratives are situated within particular places and through time. We also consider the role of agency and imagination in shaping past experiences and future selves through migratory experiences. Through this analysis, we develop insights into how our participants, who entered the UK at different times and *via* different routes, recount and make sense of experiences within and between various border crossings. Moreover, drawing on our novel dataset of diverse Afghan participants, including recent evacuees, and public discourse on border crossing our paper advances understanding of how migrants from the same origin country may be constructed differently within shifting immigration regimes. In so doing, and in keeping with the themes of this Special Issue, our paper contributes toward challenging simplistic discourses about “good”/deserving migrants vs. “bad”/undeserving migrants. In the next section we present the background context with specific focus on UK immigration policies and discourses.

## Background context

Much has been written in recent years, especially following the so-called migration crisis of 2015, about how governments have constructed migration as a “threat”, provoking a “moral panic” about the risks to security, economies, and the cultural identity of particular nation-states (Mainwaring and Brigden, 2016; Triandafyllidou, 2019; Griffiths and Colin Yeo, 2021; De Jong, 2022).

In order to understand current events, it is necessary to explain, briefly, the political context over more than a decade of UK migration policies. The notion of border as a limiting space becomes relevant in the context of the harsh austerity programme and the privatization of the welfare system initiated by the government after the 2008 financial crisis. Faced with the loss of power on the global stage, the Conservatives, elected to power in 2010, presented themselves as guarantors of citizens' security, promising a rational distribution of the increasingly scarce public resources strictly among those who paid their taxes. This message enhanced hostility toward the vulnerable and marginalized, especially irregularised migrants, as unnecessary expenses for the system (Mayblin, 2019).

Through the “hostile environment” migration policy, devised by then Home Secretary Theresa May in 2012, the Conservative administration expressed its intention to do whatever possible to expel unwanted migrants. Thus, as discussed elsewhere in this Special Issue (see paper by Wemyss, 2023) tracking and control mechanisms at the borders, as well as beyond points of entry, in everyday interactions, were intensified (Yuval-Davis et al., 2019).

In 2015, then Prime Minister David Cameron used emotive language to label the growing number of migrants crossing through the Eurotunnel a “swarm”, a term which serves to dehumanize, emphasizing the danger and threat posed by this invading force (Taylor et al., 2015). In 2018, then Home Secretary Sajid Javid questioned whether those arriving *via* irregular routes were “genuine” asylum seekers (Campbell, 2019). Meanwhile, in 2019 Prime Minister Boris Johnson proclaimed: “We will send you back… If you come illegally, you are an illegal migrant” (Quinn, 2019).

It is interesting to note, therefore, that amid severe restrictions on the rights of asylum seekers in the Nationality and Borders Act (2022),^1^ the UK government presented the Afghan evacuation plan as a humanitarian operation to save the lives of those who worked with the British Army and government in Afghanistan. The language used by leading politicians was overwhelmingly positive. For example, then Home Secretary Priti Patel said: “We owe a great deal to the brave Afghans who worked alongside us and we want to make sure they have certainty and stability to be able to thrive in the UK” (Gov.UK, 2021). Likewise, then Prime Minister Johnson said: “I am determined that we give them and their families the support they need to rebuild their lives here in the UK” (Gov.UK, 2021). Moreover, the government guaranteed evacuees' security, immediate and indefinite residence permits, medical care, and school places for children. About half of the new arrivals joined the Afghan Relocations and Assistance Policy (ARAP) resettlement scheme, which aims to provide stability with unrestricted work permission and the option to apply for British citizenship. Other evacuees joined the ACRS as Afghans vulnerable to human rights violations, such as women and girls, members of ethnic and religious minority groups, and LGBTQ+ people. However, at the same time, the Johnson government continued its policy of hostile environment toward Afghans arriving *via* irregular routes including planned off-shoring to Rwanda. Indeed, it was noteworthy that Afghans were among those on the plane scheduled to take the first group of migrants to Rwanda in summer of 2022 (Culbertson, 2022).

As explained in our Methods section, a unique aspect of our project is the diversity of participants. As well as Afghans who were safely evacuated from Kabul airport, we also interviewed some who arrived *via* irregular routes in 2021, and others who made hazardous journeys in the 1990s, before the advent of mobile technologies, and in 2015, during the so-called “migration crisis”. This novel dataset allows us to explore the range and diversity of migrant journeys *via* “irregular routes” including slow, dangerous and protracted experiences over many years, as well as the rapid movement of people through a “regular route” during the highly publicized evacuation process in August 2021. As explained in the next section, we analyse our data using “journey” as a narrative device, to gain insights into how these diverse migrants describe, present and make sense of their experiences of leaving Afghanistan and moving to the UK at different times and through different routes.

## Journey as a narrative device

The notion that millions of migrants are “on the move” and heading for European countries has become a vociferous political discourse which simplifies and misrepresents diverse migration flows (Schapendonk et al., 2021). The notion of “transit migration” has been constructed through policy discourses to present linear migratory movements (e.g., Home Office News Team, 2021) whereby migrants set out to target clear, fixed destinations, in Western Europe, and merely pass through in-between or transit countries as part of their pre-planned journeys (Crawley and Jones, 2021).

The reality of migration journeys is far more messy, uneven and uncertain (Gough and Gough, 2019; Belabbas et al., 2022), subject to changing state and socio-economic conditions, as well as migrants' personal dynamics and affective engagements at different points (Amrith, 2021). Migrants often flee dangerous and violent places without a clear plan in mind or plans may change as new routes open up or other routes close down (Koikkalainen and Nykänen, 2019). Journeys may be marked by long periods of immobility, either deliberate or enforced (Sanò and Della Puppa, 2021; Crawley and Kaytaz, 2022). Hence, as argued by Schapendonk et al. (2021), rather than always focusing on movement, it is also important to pay attention to immobility and the ways that migrants mobilize resources and navigate opportunities and obstacles in particular places where they spend extended periods of time.

Indeed, far from being merely “transit” countries, places such as Iran and Turkey, for example, are often destinations in their own right for large numbers of migrants who have no intention of ever traveling to Europe (Kaytaz, 2016; Fischer, 2017). Nonetheless, these countries are also spaces where migrants may experience complex social and economic realities that can lead to further onward migration (Askerov et al., 2018; Belabbas et al., 2022). We are mindful that only interviewing migrants who arrive in Western European countries, such as the UK, may reinforce political narratives that all migrants are moving toward this destination, the so-called politics of invasion (Mainwaring and Brigden, 2016). Nonetheless, as discussed later in the paper, our participants narrate very different migration patterns as some spent many years in other countries before arriving in the UK.

In seeking to understand complex, diverse and uneven migrations, a number of researchers have begun to use the notion of journey as “a narrative device”. Using journey as an “analytical tool”, Kaytaz notes: “the journey can be conceived as a form of narrative and that this narrative is constituted of long periods of immobility punctuated by shorter instances of travel. Conceptualizing the journey as such helps transcend the traditional, dichotomous view of the journey as having a beginning and end or an origin and final destination” (2016, p. 285). Using “journey” in this way allows researchers to analyse how migrants “construct meaning, subjectively and collectively” (Kaytaz, 2016, p. 287).

Narratives are “interpretative devices through which people represent themselves, both to themselves and to others” (Mason, 2004, p. 165). Narratives perform personal work by spelling out who I am and how I relate to others. Of course, that is not to suggest that a narrative can be understood just as an individual story. As Mason observed in her analysis of mobility stories “a gaze of individualization… loses sight of the connectivity of social relations, identity and agency” (2004, p. 178). She adds that migration narratives “are highly relational” (2004, p. 177). Personal narratives are “grounded in changing webs of relationships” demonstrating “the significance of context, contingency, constraint and opportunity” (Mason, 2004, p. 166).

Thus, it is important to contextualize narrative form, structure, content and meaning in specific spatio-temporal frameworks (Ryan, 2015a, 2023; Erel and Ryan, 2019). One does not construct narratives purely of one's own making, personal stories interact with and are shaped by wider contexts including political and policy discourses circulating in society (Ryan, 2015a). Applying a spatio-temporal lens, focuses attention on how migration is framed by the particularities of specific places and time periods. Migrants move across different places and through time, as well as negotiating a particular place over time. For example, regulations within specific jurisdictions can enable or hinder mobilities (Sanò and Della Puppa, 2021). Moreover, such regulations can shift over time, for instance in response to specific political or economic priorities, which can open or close migration pathways (Sanò and Della Puppa, 2021).

A spatio-temporal lens can be applied across various levels of analysis (Erel and Ryan, 2019). Beyond the macro structural level, we can also focus on the dynamic interplay of individual agency and inter-personal relationality, on the meso level. Hence, we analyse how migrants mobilize resources through social networks in particular places and at specific times to help navigate obstacles and opportunity not just at national border crossings but in encounters with everyday bordering within countries (Yuval-Davis et al., 2019). Thus, analyzing journeys as a narrative device, through a multi-level spatio-temporal lens, can help us to challenge the often passive or victimized depiction of migrants within public discourses (Schapendonk et al., 2021; Crawley and Kaytaz, 2022).

Furthermore, we argue that a spatio-temporal lens can also be applied to how journey narratives are presented within interview encounters. In other words, how a participant presents their narrative to a researcher is framed by the specificities of that particular spatio-temporal setting. Some participants are relating events that occurred many years earlier and now, in a place of secure status, they reflect back on their journey. In other words, they know how their journey story ended. Other participants are relating recent events from a position of unresolved status and, thus, they do not yet know where and how their story will end. As discussed later in the paper, the spatio-temporal setting of the interview encounter has implications for how journeys have been imagined and re-imagined.

The key role of imagination in how migrants see themselves in a future time, place and social setting has been underlined by Koikkalainen and Nykänen (2019) in research with Iraqi asylum seekers in Finland. In analyzing why so many Iraqis applied for asylum in Finland, a country with which they had no prior link, the researchers highlight the salience of how particular countries are constructed in individual and collective imaginations. However, upon arrival, this imagined destination often failed to live up to expectations and migrants had to begin to “re-imagine” a different future. Similarly, as noted by researchers working with Syrians in Denmark, “the futures they had imagined for themselves are not coming into fruition as they are constantly disrupted” by the bureaucracies of unwelcoming immigration regimes (Gough and Gough, 2019, p. 97). Amrith refers to how social media shapes initial linear imaginaries for migrants, generating “images of “making it” overseas, portraying life abroad as glamorous, and following a straightforward, linear story of adventure and success” (2021, p. 133). Amrith observed that even when there are rumors about difficulties, these do not deter migrants from continuing their plans.

Therefore, in applying the spatio-temporal lens, we are mindful of how stories are told retrospectively, sometimes many years later, and hence how the imagined future may have changed and been re-imagined over time. Moreover, through our novel dataset, we can also see how recently arrived Afghans, including evacuees as well as those who used irregular routes, are in the process of imagining and re-imaging their future selves in an evolving context. In the next section we present a brief summary of our research methods and dataset.

## Research methods

This article draws upon a multi-methodological and intersectional study that took place in January–July 2022 and involved 30 newly arrived and long-established Afghan migrants in London. The project received ethical approval from London Metropolitan University research ethics committee.

As a relatively small qualitative study, we make no claims to representativeness, nonetheless, consideration was given to the diversity of the sample in terms of gender, age, family situation and time of arrival. Just over half of our participants (56.7%; *N* = 17) identified as female, and 43.3% (*N* = 13) identified as male. Route and year of arrival in the UK varied greatly among participants, with just over half (53%; *N* = 16) having arrived between 2020 and 2021. All participants have been pseudonymised, with culturally appropriate names, to protect their identities.^2^

All interviews were carried out by the two authors. Interpreters were offered where needed but most interviews were conducted in English either because participants had already lived in London for several years and were comfortable to be interviewed in English or because, though recently arrived, they were highly educated and spoke English confidently.

Interviewing refugees involves specific ethical considerations especially because of the trauma they may have experienced and because of concerns they may have about answering questions relating to their status and route of entry. We are reflective of positionality and power dynamics within the interview encounters (Ryan, 2015b). Access was negotiated through two Afghan associations^3^ and the peer researchers, who were all Afghans.^4^ All interviewees and focus groups participants were offered a voucher for £20 for their time. Likewise, the four community-based peer researchers received training and were paid vouchers as a recognition of their time on the project. But as noted by Miller (2004) access is not the same as trust. The research project was clearly presented as a collaboration between the university and the Afghan associations. Most interviews took place in the association premises or in hotels where the associations were regularly visiting. Thus, our clear links with Afghan groups probably helped to assuage some potential concerns about our research. Moreover, most of our participants were university graduates or had worked at universities and hence had a good understanding of how university research operated. Nonetheless, we are aware that participants may not have shared all their stories with us (Miller, 2004). However, by focusing on “narratives” we are taking account of these as the versions of their stories that participants felt comfortable sharing with us (Ryan, 2015b). Indeed, as Kaytaz notes, constructing narratives is an essential skill for immigration procedures “particularly for the asylum process” (2016, p. 294). Hence, we are mindful that the narratives of their migration journey that participants shared with us in the interview encounters may be shaped, at least in part, by how they have developed that particular account over time as they navigate immigration and asylum procedures. As Azarian notes, “a story is primarily a justifying narrative” (2017, p. 692) which seeks to explain and rationalize a particular line of action.

All interviews were fully transcribed and anonymised. Both authors, and the research assistant Alessia Dalceggio,^5^ undertook initial thematic coding of each individual transcript, based on our original research questions and literature review but also allowing new themes to emerge. We then developed a detailed coding tree and the dataset was entered into the software package NVIVO which is suitable for the analysis of qualitative datasets.

The 30 Afghans who took part in our project had arrived in London at different times and *via* different routes. In this paper, because we wish to explore the journey as a narrative device, we have decided to use a case study approach and present five journey narratives in some detail. Applying a multi-level spatio-temporal lens, our five participants illustrate different journeys, across diverse routes, and at varied points in time, with a range of examples of periods of immobility in particular places. Rabiya fled Afghanistan during the first Taliban regime in the 1990s and spent years in the Netherlands before moving to the UK. Bilal lived for many years in Iran before traveling to the UK in 2015 at the height of the “migration crisis”. Both Malala and Abubakar were part of the evacuation from Kabul in August 2021 but illustrate different experiences of that process. Having missed out on evacuation, Sher Shah arrived in the UK *via* irregular routes in 2021.

## Migrants stories of using irregular routes

### Rabiya

Rabiya was interviewed in person in an Afghan community organization. Her narrative began with her working as a pediatrician in a children's hospital in 1997, when the Taliban came to power. She said: “They entered the hospital with guns”. Rabiya stated that it became impossible for her to continue working: “Also, my father worked in the government before Mujahedeen came to power… so I felt more in danger… I was not a politician, but I studied medicine in Russia, and they did not like people who studied in Russia; they called us communists”.

Rabiya then described how, in 1997, she fled Afghanistan by car with her young son and husband. She told us about crossing into Uzbekistan and that they stayed in a room for three weeks before traveling to Tashkent by train to avoid passport controls. Throughout her journey narrative, across international borders, Rabiya highlighted active agency in negotiating risks and mobilizing networks at risky situations, thus rejecting the role of victim or dupe (Schapendonk et al., 2021). Although there was a tendency in her narrative to brush over details about any dangers and uncertainties, she did mention that she wore a burka for fear of being arrested: “It was my first time and was my last time to wear a burka. I threw this away [laughing]”. Describing how the simple act of wearing a burka seemed to mislead the soldiers at the checkpoints along the road, suggests Rabiya's sense of satisfaction in subverting the very item of clothing so inscribed with symbolism in Taliban-controlled Afghanistan.

Although it was clear that the long journey over land, involving cars, lorries and a bus relied largely on people smugglers, Rabiya gave us few details but simply said that her husband made all the necessary arrangements. Nonetheless, she did describe the risks they encountered as they endeavored to cross the Polish border: “it rained a lot on the Polish border, and it was flooded; we had to get a plastic boat”.

The family was trying to get to the Netherlands, where Rabiya's sister lived. The spatio-temporal context of the narrative is highly significant as it took place in the late 1990s before the widespread use of mobile phones. Unlike recent migrants who rely so heavily on mobile technology (Gough and Gough, 2019), Rabiya did not even have a telephone number for her sister and so was unable to communicate or even notify her that the family were on their way to the Netherlands.

Rabiya did not tell us much about the uncertainties and intermittent waiting along her winding journey through Poland and Germany. While the family clearly relied on traffickers, Rabiya was quite “matter-of-fact” in her account. Here is it important to acknowledge the role of memory as she recounted events that had taken place over 20 years earlier and when the desired outcome of safely reaching the Netherlands had been achieved. Thus, it is possible that she remembered and presented the journey as successful and hence downplayed the risks, fear and costs. Her narrative focused on the positive side of her life in the Netherlands, first in a refugee camp set up in a “big land with a big ground; all green”, where there was “a lot of caravans for refugee people” and where the family lived for more than a year. Rabiya focused on the facilities provided in the caravans, which she described as a home-like space. One year after their arrival in the camp, the family obtained their legal status and moved close to where Rabiya's sister lived, where they stayed for 10 years. Rabiya described how neighbors welcomed her family and told us about her efforts to navigate the structural constraints for migrants and her attempts to learn the Dutch language. While she was positive about many aspects of Dutch society, she also described experiences of anti-immigrant hostility from some neighbors.

Although Rabiya and her family spent 10 years in the Netherlands and attained Dutch citizenship, she said that she always dreamed of moving to the UK in search of better opportunities for her children: “If they know good English they can find a job everywhere because it is an international language, while Dutch is just used in 300 km”. Hence, in 2007, the couple and their two school age children arrived in London. Again, the spatio-temporal framing of her migrant journey is significant because, at that time, prior to Brexit (Britain's departure from the EU), their Dutch citizenship ensured their EU mobility rights so they crossed the border to the UK without any restrictions.

Rabiya's expectations for her children in the UK show the significant role of imagination in migrants' decision-making on their journey (Koikkalainen and Nykänen, 2019). Nonetheless, while feeling settled in London with her family, Rabiya reflected her disappointment with the UK system for not allowing her to validate her doctor's degree—despite her many volunteering jobs related to healthcare. It is apparent that the lack of recognition of her degree had failed her expectations regarding coming to the UK: “I can't do anything [sigh]… Finding a job is important…” Rabiya clearly felt frustrated by her inability to re-start her medical career in the UK. Nonetheless, she wished to present her journey in a positive light. As noted, a narrative may be a justification story seeking to make sense of a particular set of actions and decisions (Azarian, 2017). Hence, her narrative ended by asserting that “it is more important to be healthy and have my family with me”. Like many migrants who experience de-skilling, she emphasized the educational and employment achievement of her children and thus her migration journey could be presented as a success.

### Bilal

We interviewed Bilal at an Afghan association. His story began when he was a young shepherd in a village. Tired of poverty, he decided to move to Iran with friends in the 1990s. Bilal described how in Iran, where he stayed for 20 years, he suffered discrimination for being Afghan. Despite speaking Farsi and being a Muslim, his migration status and Hazara ethnic background marked him as an outsider in Iran. He recounted numerous incidents of being stopped by the police and being asked to show his documents. Thus, beyond the crossing of actual physical borders, “everyday border guards”, like the Iranian police in Bilal's narrative, continue to monitor the movement of migrants (Yuval-Davis et al., 2019). Bilal's narrative of ethnic discrimination in Iran offers another example of how political policies create “normative artefacts” (Schapendonk et al., 2021). Although much needed manual workers, Afghans were continually harassed and discriminated against within Iran (Kaytaz, 2016; Crawley and Kaytaz, 2022). Nonetheless, Bilal stayed there for 20 years, married and had two children. Thus, Iran cannot be simplified as a “transit” country through which Bilal passed on his journey to the UK. His story illustrates how his journey involved extended periods of immobility (Sanò and Della Puppa, 2021). Eventually, seeking a better life for his young family, he decided to move with friends to Turkey, with the help of smugglers, whilst his wife and children waited in Iran.

He became vague at providing details about how he got in touch with the smugglers but highlighted the risks he faced while crossing the Iran–Turkey border through the mountains. Bilal became emotional as he revealed that migrants did not differentiate between security forces, immigration officials and smugglers, as they all posed a persistent threat in such a dangerous context: “On the mountains, you encounter many thieves; we don't know if they are from the government, thieves, Turkish civilians… who are they? I don't know”.

While interviewing Bilal it was obvious that he was disabled and used a wheelchair. We had assumed this was due to some recent illness or accident because, up to this point in the interview, while describing his journey, he never mentioned the wheelchair. We simply asked at what point his accident occurred as he now uses a wheelchair. To our surprise, he explained that he always used a wheelchair, since childhood, and his entire journey had involved the wheelchair. This incident gave us a new perspective of Bilal's journey experience, revealing how migrant journey narratives may involve taken for granted assumptions that are not explicitly stated or explained in interview encounters.

At our request, Bilal then retold the story of how he had managed to cross the Iran-Turkey border in the wheelchair. It was only at that point that he provided a detailed account of being tied to a horse: “When I said I could not keep myself on the horse, they did not care”. He almost broke down in tears when recounting that one of his most tragic experiences in the mountains was the sight of dead bodies who had been shot by the Iranian police: “We just… you don't ask anything”. He acknowledged that although he cried when he saw the bodies, the traffickers did not halt for fear they would also be shot: “They [the Iranian police] don't care if you are disabled for example, or you are woman; they don't care. They will shoot you”. At that point, Bilal reiterated his lack of trust in those he paid for crossing the mountains and mentioned that he carried a weapon to defend himself: “You pay money but maybe your money has gone, and your life goes too”. Bilal's expressions of distrust highlighted the complex relationality underpinning journeys through illegal routes. He placed his life in the hands of people that he did not trust and who might also pose a threat to his survival.

While he felt less discrimination in Turkey than in Iran, Bilal explained that he and his friends ran out of money and had to sleep in parks. Bilal related an anecdote of meeting a man in a park. The man told Bilal that almost his entire family, except himself and his little daughter, had drowned while trying to reach Greece by boat. Nonetheless, the man stated that he was determined to try again. Throughout Bilal's narrative it is apparent how migrants' collective construction of potential rewards awaiting them beyond Greece motivated the use of highly dangerous routes, with no pre-established plan, or guarantee of success but with the acknowledged risk to their lives (Koikkalainen and Nykänen, 2019). As Bilal explained:

It is a long process along which most people die. Many people die when they cross from Afghanistan to Iran, but if you stay in Afghanistan, you will die too. Most people don't feel safe; every day you get attacked, there is an explosion, a bomb… But then you are not safe in Iran. So you move to Turkey, and find lots of problem too.

After the conversation with the man in the park, Bilal decided to change his strategy and get a false passport to enter Germany. He was reluctant to explain the process of acquiring false documents and we did not probe. From Germany he crossed to Belgium and then France. He described his experience at the Calais Jungle, where he stayed for 3 months. He underlined the violence inflicted upon migrants by the authorities and lack of institutional support. He explained how people shared stories of attempting to cross to the UK by boat, train and even freezer lorries, which he said he was about to try but he was discouraged from doing so. While his narrative, to some extent, presented an individual endeavor, it is also apparent that relationality was a key enabling factor in his journey. As noted earlier, journeys are narrated as subjective but also collective experiences (Kaytaz, 2016) and it is clear that migrants he met along the way shared information and advice on the risks and viability of particular routes (D'Angelo, 2021). While Bilal was reluctant to tell us about how he finally crossed into England with the help of smugglers, his narrative clearly presented the intricate dimensions of his long and protracted journey *via* illegal routes.

In the UK, he claimed asylum and eventually he got residency a year later. Having secured his status, he was able to bring his wife and children to the UK. His wife is now training in accountancy and he is proud that his children have settled well into school in London. Thus, having lived in the UK for more than 5 years, he presented his migration journey as successful and realizing his dreams.

### Sher Shah

We interviewed Sher Shah *via* video link from his bedroom in a “contingency”^6^ hotel in London. A 26 year-old student, he fled the Taliban in 2021 but he was unable to reach Kabul airport and so was not part of the evacuation. Instead, he used irregular routes to come to London. His narrative provided detailed accounts of his journey over several months and through numerous countries. Unlike Rabiya and Bilal who did not initially intend to move to the UK and who stayed for many years in other countries, Sher Shah had intended to get to the UK as quickly as possible.

His narrative began with his father paying smugglers to take Sher Shah and his wife to Turkey *via* Iran by car and lorry, with a group of other migrants. They started their journey on 30th August 2021: “It was the first time that I came out from my country, and it was difficult”. In Turkey, Sher Shah described how he and his wife traveled by minibus with a group of five others: “There were different prices, but I think it was around $2,000 per person [from Iran to Turkey], and we had to pay $4,000 or maybe $5,000 because we were two people”.

He became emotional as he narrated an incident that occurred in Turkey. One day having gone out to buy food, Sher Shah returned to the hotel to discover that his wife was gone: “At first, I did not know what had happened. Nobody told me”. Later he found out that the Turkish authorities had raided the hotel: “In that hotel there were lots of people from Afghanistan and other places; they took all of them”. Sher Shah could not go to the authorities enquiring for his wife as that may mean his own arrest. A few days later he received a phone call from his wife to say that “she had been deported to Iran”. His wife persuaded him to carry on without her: “It was hard… she told me not to come back and that there may be hope for me and the family”.

Fearful of being arrested and deported, Sher Shah described how he stayed hidden for around 2 months until he was able to hire the services of people to help him continue his journey to Italy: “I knew some people from Afghanistan in Turkey, but they didn't help me”. His story suggests that in such dangerous and difficult contexts co-ethnic networks may not be willing to share resources (D'Angelo, 2021).

Throughout his narrative it is apparent that he relied on smugglers to get across national borders, but he was reluctant to tell us how he had contacted those people. While he understood that we were university researchers, it is likely that he was still distrustful of sharing certain details with us. The spatio-temporal context is relevant here because, unlike Bilal and Rabiya who had secure status, Sher Shah was still going through the asylum process. Nonetheless, he appeared to be remarkably open and shared a lot of information in his interview. As noted by Triandafyllidou (2019) in her research in Greece, migrants often discuss their use of smugglers as a taken for granted necessity in facilitating migratory journeys.

Sher Shah described traveling to Italy by boat with approximately 72 people of different nationalities. It took “four days and four nights” to reach the Italian coast: “If someone had intercepted us… they would have sent us back to Turkey”. In Italy, they traveled by lorry and train. Then they split into smaller groups, and Sher Shah went with four other people to France. The group spent around 15 days in the Calais Jungle without any support such as fresh clothes, food or a roof over their heads:

We spent several nights in the rain. It was difficult for us. We had nothing and we stayed there in the jungle without anything, not a place. Then slowly, slowly, we found some help: there was an organization that helped people in Calais, and they helped us; they gave us a tent and some clothes.

In the Jungle, Sher Shah continued to rely on smugglers that his father paid at different points along the way. It was not clear to us if these were the same smuggler organization or different groups in specific countries. Sher Shah became vague when we asked about the smugglers, but he did tell us the varied prices: “From Turkey up to Italy, I paid around €8,500… Lots of money”. The smugglers arranged his journey from Calais to Dover by lorry. He narrated the various attempts to enter UK. At first the smugglers tried to get three other people in the back of a lorry, but the police intercepted them and sent them back to Calais. The smugglers then decided it would be safer for Sher Shah to travel alone. He paid £3,000 for this leg of the journey.

Once in the UK, the lorry carrying Sher Shah traveled for several hours. Alone in the back of the lorry, he underlined his feeling of powerlessness and disorientation because his mobile phone battery had died. He was completely out of contact with anyone and was unable to track his location. Eventually the lorry stopped: “I jumped out, and I didn't know the place. It was not Dover… It was near London some place”. Sher Shah described walking for about an hour and reaching a small train station. “I asked someone: how can I go to London? And he told me to take this train to London”. In London, Sher Shah approached a policeman: “I told him that I came to live here, to help me if it's possible, because I don't know anything here”.

Police officers arrested Sher Shah because he had crossed the border illegally: “The whole time was difficult for me. I was scared from the beginning to the end, up to when I went to the police station… and I was very afraid of that place too. But everything has passed here… It was so hard for me”. With no knowledge of the system, he claims: “It was the hardest night of my life, so hard for me”. At this point, Sher Shah interrupted the story, he moved away from the camera and we were unable to see him. He said he needed a break. Because the interview was taking place by video platform it was difficult for us to comfort him or to see how upset he had become. After some time, he moved back to the camera, looking visibly upset, but agreed to continue, though we offered him the opportunity to pause or stop. He seemed keen to tell his story, though he did not wish to elaborate on what happened at the police station.

He claimed asylum on 10th December 2021 and was sent to a contingency hotel in South London. Since then, he has been relocated outside London. We have emailed to him to know how he is doing but he has not replied.

Although Sher Shah was fleeing the Taliban at the same time as those evacuated from Kabul airport, his journey to the UK, treatment upon arrival and route to asylum are very different from the evacuees. As noted at the start of the paper and illustrated by our two examples below, Afghans who were evacuated directly from Kabul airport are on a direct route to secure status and hence are treated very differently from those who fled the same situation but arrived in the UK *via* irregular routes. Sher Shah tried to go to the airport but was not successful. As discussed below, there was a randomness to who was evacuated from Kabul and who was not. Sher Shah's story illustrates how immigration regimes create “normative artefacts”, categories of deserving and undeserving migrants, even within the same national and ethnic groups.

## Evacuees' stories

### Abubakar

We interviewed Abubakar at a so-called “bridging”^7^ hotel, in central London, while he was waiting to be re-housed. Prior to his evacuation, Abubakar informed us, he had held a senior level government post in Afghanistan. The sudden collapse of the government in August 2021 had come as a shock, he explained.

He described witnessing tanks pouring on to the streets, to the astonishment of the general population and to government officials like himself. He recounted how the Taliban rebuked him: “You have a very big Land Cruiser, bullet-proof car, and this belongs to the government”. Abubakar explained the risks associated with being identified as a government official and how he had tried to conceal his role from the Taliban soldiers. He narrated the conversation: “See my document, this car does not belong to government; it's mine”. A Taliban commander had then said: “You don't have a beard”; Abubakar replied: “I don't have it”, and the commander slapped him. In this short but powerful exchange, Abubakar conveys his sense of danger, given his former position within the government, and the risks posed to him by the incoming Taliban regime.

He shared another anecdote with us to underline the rapidity with which the situation changed and the growing danger for him: “When I was living in Kabul, in front of my house there were some guys selling tomatoes and cucumbers in a cart. When I came to my house, those guys had guns and used turbans. Everywhere I went, I heard: ‘Oh, you work for the government, we will kill you!”'.

Desperate to leave Afghanistan, Abubakar described quickly mobilizing his connections with the British authorities in Kabul. On 18th August 2021, he sent a WhatsApp message to the British Embassy asking for help to leave: “my life is not safe, I want to go somewhere”. A week later, the British Ministry of Defense asked for his full name and passport number and sent him an email with instructions to go to the Baron Hotel, near the airport, “as soon as possible”.

Abubakar described the scene as he approached the processing center: “a lot of rush and scrabbling of people'. Despite his former rank and authority, he now needed to join the queue alongside thousands of other Afghans. Waiting in the queue for eight hours, Abubakar recounted that a man organizing the queue asked with surprise: “‘What are you doing here?”' I said: “What is everybody doing here? We all want to leave Afghanistan”. “Do you have any approval, like an email?” I said: “Yes, I have my email”. “Please come to the gate”.“ This encounter seems to imply that the official was surprised to find a high-ranking government official in the queue (*what are you doing here?*) and quickly moved Abubakar's inside the processing center. Rank and connections can facilitate the processes of crossing borders. After a very short time, he was on the plane and on his way to the UK. The evacuation, although chaotic and highly dangerous, as mentioned at the start of the paper, was also relatively quick. In contrast to the long, protracted journey of people like Sher Shah who traveled for months *via* Turkey and Greece to France and finally to the UK, evacuees found themselves traveling from Kabul to London within 24 hours.

Upon arrival in London, Abubakar narrated his continued efforts to mobilize his connections through personal social ties. He made contact with a British politician whom he met in Afghanistan. However, despite repeated messaging, so far, this individual had not been available for a meeting. This situation is quite interesting because it is apparent that Abubakar had successfully mobilized his contacts to expedite his passage out of Kabul, at a time when many thousands of Afghans were unable to do so. However, once in the UK and now a refugee, rather than a senior government official, it seemed to be harder to mobilize these influential connections highlighting barriers to accessing “vertical social ties” (Ryan, 2016).

Although Abubakar was keen to assert his status, and presented us with his recently printed business cards, it was also clear that his imagined future in the UK had started to undergo re-evaluation. While stating his wish to work with the UK government in some advisory capacity, nevertheless, he seemed mindful of the struggles and disappointments that may be encountered in realizing his imagined future. His cousin in the UK works as a taxi driver. Abubakar recounted conversations with other refugees in his hotel in which they advise each other: “don't work in the Pizza Hut and minicab in Uber”. Even by mentioning such jobs, Abubakar indicated his concerns about the possibility of becoming trapped in low skilled, low paid work. Thus, he tried to preserve his dream of a government job, while watching other Afghans become Uber drivers.

Another potential threat to his imagined future is the public perception of refugees. At the moment he perceived British society as welcoming of Afghan refugees and he described everyday interactions when people say “you're most welcome”.

Nonetheless, Abubakar expressed concerns about other Afghans arriving in the UK *via* irregular routes.

“[Before the Afghan government collapsed in 2021,] there was no poverty: we had food; everybody had a job. There was no poverty. In that time, nobody wanted to come illegally to the UK. When the government collapsed, the international community, especially the UK government, decided to help the Afghan people because some of them had very good relationship with the British government and the embassies. So they decided to bring these kind of people to the UK. Before 2021, refugees came to the UK illegally without permission of the government. Afghan refugees are most welcome by the UK government because of the approval of the Parliament”.

In this way, Abubakar clearly sought to distance himself, a “genuine” refugee, who is on track to receive full status in the UK, in contrast to Afghans who had entered the UK “illegally”. He calls into question the legitimacy of their claims for refuge. In so doing, he appears to completely accept and reproduce the UK government discourse that distinguish between “deserving” and “undeserving” refugees.

### Malala

We interviewed Malala online, from her room in a bridging hotel, though we later met her in person during several dissemination events. Malala (23) and her two sisters (25 and 26) arrived in the UK as part of the evacuation in August 2021. Malala told us how her father passed away when she was seven years old, and, several years later her mother had left Afghanistan to join a brother in another country. Thus, Malala and her sisters were living in Kabul “without a male guardian”. When the Taliban took control of Kabul in August 2021, Malala was a university student, studying journalism and computing, with dreams of setting up an all-female IT company: “I studied Computer Science and saw how women struggle to find a job because in Afghanistan the belief is that women are not good at IT. I wanted to set up a company and hire female employee and challenge the status quo”. However, her imagined future was destroyed when the Taliban took control of Kabul.

Malala's narrative powerfully evoked her shock when the government suddenly collapsed on 15th August 2021. Her sister had worked in a government department, moreover without a male guardian the three siblings felt vulnerable: “Everyone was scared. We hide our ID cards because I was a University student and a journalist, which were both very dangerous”.

In an effort to escape from Kabul, Malala told us how she quickly mobilized her social capital by enlisting the help of a journalist from a well-known European newspaper. Malala had gotten to know this journalist by assisting her on some stories in Afghanistan. “We were in touch all night and day. We were hopeless that it might work because the situation was bad at the airport”.

The British Embassy responded on the last day of the evacuation, 26th August, and the three sisters headed to the airport. They tried to convince the authorities that their emails were authentic while standing for hours in a filthy canal: “The men didn't trust us… They just looked at our documents and handed them back to us”. Malala told us that many people were using faked IDs or claiming people as their relatives in order to secure their evacuation, while “so many other people who deserved to be evacuated were left behind”. In the chaos at the airport, Malala asserted the seeming randomness of who was evacuated and who was left behind: “I was so shocked, because there were a lot of people…and they were not at risk at all, and they were allowed to go through”. She told us about an Afghan policewoman who was in danger from the Taliban but, without influential connections to support her claims, was unable to navigate the evacuation process and was left behind. Thus, in contrast to Abubakar who positioned himself as a deserving refugee unlike those arriving “illegally”, Malala presents a more nuanced picture that suggests the apparent unfairness of who achieved evacuee status.

Malala vividly described the dangerous scene at the airport. After waiting for hours she finally caught the attention of two British female soldiers: “They found me in the water; I handed the emails to them, and then they checked my sisters' names, and they allowed us all to get in”. The British Army checked the sisters' IDs and interviewed them because they did not have British passports. They then checked their identities by phoning their influential contact, the well-known journalist, and the British Embassy, and finally gave the sisters permission to leave Kabul.

Malala and her sisters traveled on a military plane from Kabul to Doha, where they stayed for one night, and then were put on a commercial flight to London. Malala recounted her mixed feelings:

“It was kind of joy that I was safe, and it was sad that I was leaving my country behind and lots of friends behind… I was worried about everyone”.

After their plane landed at Heathrow, they were hosted in a quarantine hotel for 10 days and then moved to a bridging hotel in Central London, where they remained for over 1 year. Having suddenly found herself in London without any prior plan to leave Afghanistan, Malala, like many other evacuees, is having to rebuild her life, re-define her dreams and re-imagine her future in the UK. Unlike those who embark on long journeys where much time is devoted to imagining a new life in the destination society (Koikkalainen and Nykänen, 2019), rapid evacuation offers little time for refugees to imagine what their new lives will look like. Malala and her sisters had no plan to move to London. Their imagined future involved study and work in Afghanistan. Now, they find it hard to even imagine a return to their homeland while the Taliban is in control. Malala explained how she was embarking on a new course of study and trying to restart her life and regain her dreams in London. Unlike those who are contained within asylum processing centers (Gough and Gough, 2019) or in contingency hotels like Sher Shah, awaiting their fate, Afghan evacuees in London have the right to access education and training. Malala concluded: “I feel so lucky that I am here, otherwise my sisters and I were alone in Afghanistan, and God knows what would happen to us”.

## Conclusion

In this paper we have presented rich case studies drawn from our diverse Afghan participants. A novel feature of our research is that it included Afghans who arrived in the UK over many decades from the 1990s up to the 2020s *via* a range of different routes. The inclusion of recent evacuees has allowed us to consider how their experiences differ not only from earlier arrivals but also from Afghans currently arriving *via* unofficial routes.

Engaging with the concept of “migrant journey” (Amrith, 2021; Gough and Gough, 2019; Crawley and Jones, 2021; Schapendonk et al., 2021) and using journey narratives as an interpretative device (Mason, 2004; Kaytaz, 2016), we have applied a spatio-temporal lens (Erel and Ryan, 2019) to analyse how migrants' journey narratives are situated within particular places and through time. For example, we have shown the continuities but also changes in socio-political contexts as well as other infrastructures especially the use of mobile communication technologies. Analyzing journey narratives, through a multi-level spatio-temporal lens, we have sought to advance understanding of the dynamic interplay of macro socio-structural contexts, meso level of inter-personal networks and micro level individual migrant agency.

On the macro level, we have shown how different policies of the British government toward Afghans, arriving *via* different routes, has created an artificial distinction between those who are fleeing the same conflict. In marked contrast to the proclaimed welcome for Afghan evacuees, those escaping the Taliban regime *via* illegal routes are vilified as undeserving and fraudulent in public discourses and “irregularised” (El-Enany, 2020) in immigration policies with the threat of being off-shored in Rwanda. Hence, while it is known that different migration categories are “normative artefacts” produced by immigration policies (Schapendonk et al., 2021), our paper goes further by showing how this categorization can occur even within the same national grouping at the same moment in time.

On the micro level, it is apparent that, in contexts of extreme danger where migrants were confronted by the risk of death, their individual narratives present agentic qualities in overcoming repeated hazards in journeys over land and sea. However, even if traveling alone, the role of significant others, acting on the meso level between the individual and the wider structures (Ryan, 2023), indicates the key role of networks as a linking theme throughout the narratives. Those traveling *via* irregular routes relied on complex and dynamic ties ranging from enduring kinship ties to fleeting contacts with fellow travelers. However, network ties are not necessarily positive and, although essential to their journey, relations with smugglers could be exploitative and dangerous (D'Angelo, 2021). Access to networks was also a factor in navigating the chaos of Kabul airport during the evacuation process. Those with vertical ties (Ryan, 2016) to influential contacts, such as foreign journalists and embassy officials, were able to secure safe and speedy passage out of Afghanistan. By contrast, it is apparent that some people who faced risks from the Taliban, but who lacked influential contacts, were left behind. Thus, far from being fair, the evacuation process underlined privilege and power, further complicating categories of “deserving” and “undeserving”.

Finally, by applying the spatio-temporal lens to our diverse dataset, our paper also advances understanding the role of imagination in how migrants' journey narratives are told in research encounters. Our diverse participants, traveling at different times and through different routes, have offered varying insights into how future and past lives are imagined and presented in journey narratives. For some, especially those who left Afghanistan many years ago, narratives can involve a reflection on how the imagined future has been realized (or not) over time. Imagination is a key ingredient in the narrative of migrant journey not just as a story of the past but as a device for making sense of their present and expected future. By contrast, for those who recently arrived, especially those who experienced rapid evacuation and who suddenly found themselves in London, almost overnight, imagined futures are still being constructed as they adjust to new and unfamiliar environments. Moreover, those who had recently arrived *via* irregular routes are in positions of uncertainty about their future migration status, and thus their narratives present on-going journeys through asylum applications. They do not know how, when and where their story will end.

The UK policy of closing down routes of legal entry and making the asylum process more difficult, has resulted in rising numbers of people trying to enter the country through irregular routes.^8^ As noted by many NGOs, it would be more humane to assess asylum applications based on the actual risks and threats faced by individuals rather than on their route of entry.^9^ As the UK courts recently declared the planned off-shoring to be legal,^10^ there is a real risk that Afghans fleeing the Taliban, but who narrowly missed out on evacuation, will be denied any legal route to asylum in the UK and will instead face the prospect of being off-shored in Rwanda.

## Data availability statement

The original contributions presented in the study are included in the article/supplementary material, further inquiries can be directed to the corresponding authors.

## Ethics statement

The studies involving human participants were reviewed and approved by London Metropolitan University. The participants provided their written informed consent to participate in this study. Written informed consent was obtained from the individual(s) for the publication of any potentially identifiable images or data included in this article.

## Author contributions

All authors listed have made a substantial, direct, and intellectual contribution to the work and approved it for publication.

## Footnotes

^1^For more details about this Act see, https://www.legislation.gov.uk/ukpga/2022/36/contents/enacted.

^2^For more information about the project see https://www.londonmet.ac.uk/research/centres-groups-and-units/global-diversities-and-inequalities-research-centre/projects-and-partners/afghan-migrants-in-london/.

^3^We worked closely with Paiwand and the Afghan Association in Harrow, London.

^4^The four peer researchers were Najiba Askari, Khandan Danish, Farid Fazli and Samiullah Khaillyzada.

^5^Our Ph.D. student Alessia Dalceggio assisted us in this project and was particularly involved in data coding and writing the research report.

^6^Contingency hotels were used to house migrants who arrived from Afghanistan, via irregular routes, following the Taliban take over. With deportations temporarily halted and off-shoring not yet started, these were intended as short term accommodation while asylum applications were processed.

^7^Bridging hotels were usually high-end hotels, including 5 star hotels, used to temporarily house evacuees. Because of the Covid-pandemic, those hotels happened to be empty in summer 2021. Although intended to be temporary, while waiting for permanent re-housing, we found that many of our participants were still living there more than one year later.

^8^ https://www.refugeecouncil.org.uk/latest/news/why-the-governments-approach-to-channel-crossings-fails-people-in-need-of-protection/

^9^ https://www.refugeecouncil.org.uk/get-involved/campaign-with-us/safe-routes-save-futures/

^10^https://apnews.com/article/rwanda-covid-asylum-03de7919f185482ff9cef99bbf1b725d 19 Dec 2022.
